# Genomic surveillance of extended-spectrum cephalosporin-resistant *Escherichia coli* isolated from poultry in the UK from 2016 to 2020

**DOI:** 10.3389/fmicb.2023.1335173

**Published:** 2024-01-30

**Authors:** Nicholas Duggett, Manal AbuOun, Emma Stubberfield, Olivia Turner, Luke Randall, Robert Horton, Javier Nunez-Garcia, Daisy Gates, Jeremy Chanter, Chris Teale, Muna F. Anjum

**Affiliations:** ^1^Animal and Plant Health Agency, Addlestone, United Kingdom; ^2^Animal and Plant Health Agency, Thirsk Veterinary Investigation Centre, Thirsk, United Kingdom; ^3^Animal and Plant Health Agency, Starcross Veterinary Investigation Centre, Exeter, United Kingdom; ^4^Animal and Plant Health Agency, Veterinary Investigation Centre, Shrewsbury, United Kingdom

**Keywords:** antimicrobial resistance, multidrug resistance, surveillance, *Escherichia coli*, plasmids, poultry

## Abstract

**Introduction:**

Surveillance is vital for monitoring the increasing risk of antimicrobial resistance (AMR) in bacteria leading to failures in humans and animals to treat infections. In a One Health context, AMR bacteria from livestock and food can transfer through the food chain to humans, and vice versa, which can be characterized in detail through genomics. We investigated the critical aspects of AMR and the dynamics of AMR in poultry in the UK.

**Methods:**

In this study, we performed whole genome sequencing for genomic characterization of 761 extended-spectrum cephalosporinases (ESCs) harboring Escherichia coli isolated from poultry caeca and meat through EU harmonized monitoring of AMR in zoonotic and commensal bacteria from 2016 and 2018 and UK national monitoring in 2020.

**Results:**

The most common ESC in 2016 and 2018 was blaCTX-M-1; however, 2020 had a greater diversity of ESCs with blaCTX-M-55 dominant in chickens and blaCTX-M-15 more prevalent in turkeys. Co-resistance to sulphonamides, tetracycline, and trimethoprim was widespread, and there were several positive correlations between the sequence types (STs) and ESC genes. We identified certain AMR genotypes and STs that were frequent each year but not as successful in subsequent years, e.g., ST350 harboring blaCTX-M-1, sul2, and tetA-v4.Phylogenetic comparison of isolates prevalent in our panel with global ones from the same STs available in public databases showed that isolates from the UK generally clustered together, suggesting greater within-country than between-country transmission.

**Discussion:**

We conclude that future genomic surveillance of indicator organisms will be invaluable as it will enable detailed comparisons of AMR between and within neighboring countries, potentially identifying the most successful sequence types, plasmids, or emerging threats.

## Introduction

Antimicrobial resistance (AMR) is a growing health concern that may lead to failed prevention and treatment of infections, including common ones, which, if not addressed, could result in millions of deaths worldwide (O’Neill, 2016). A particular concern is resistance to extended-spectrum cephalosporins (ESCs) owing to their use within healthcare and veterinary settings (UK-VARSS, 2019; UK Health Security Agency, 2021). As such, they have been designated as the highest priority critically important antimicrobials (HP-CIAs) to humans and veterinary critically important antimicrobial agents (VCIA) to animals by the WHO and WOAH, respectively (OIE, 2007; World Health Organization, 2019). Resistance is monitored in indicator organisms, particularly *Escherichia coli*, within livestock in the EU and UK. The emergence of AMR can result from spontaneous mutation, but dissemination is primarily driven by plasmids and mobile genetic elements that can transverse bacterial species and phyla. Evidence of this spread in poultry harboring ESC *E. coli* has been widely documented, with *bla*_CMY-2_, *bla*_SHV-12,_ and *bla*_CTX-M-1_ being the most commonly identified genes that have been associated with a myriad of plasmid Inc-types (Saliu et al., 2017; von Tippelskirch et al., 2018; Aldea et al., 2022).

Whole genome sequencing (WGS) is a useful tool for detecting, understanding, and combatting the spread of AMR through increased genomic resolution over traditional laboratory-based microbiology (Duggett et al., 2020). WGS can provide detailed information about the presence of AMR genes and plasmids, speciation, and typing of the bacterial host, among countless other applications. Furthermore, it can facilitate the rapid and detailed phylogenetic comparison of isolates, resulting in the analysis of the trends of particular variants and elucidating links between isolates and their genes (Nunez-Garcia et al., 2022). Currently, the European Food Safety Authority (EFSA) allows member states to submit WGS data for the monitoring of ESCs in indicator organisms but this is on a voluntary basis (EFSA et al., 2023).

The present study aimed to perform genotypic characterization of 761 ESCs *E. coli* isolated from unique epidemiological units collected through national and EU AMR monitoring of caecal contents and meat from chickens and turkeys. Furthermore, phylogenetic comparisons were performed to establish any link between the most common sequence types (STs) harboring the same AMR genotype(s), circulating in poultry from the UK, with global isolates available from public repositories.

## Methods

### DNA extraction of *Escherichia coli*

Chicken caecal isolates from 2016 and 2018 originated from archived *E. coli* that had been collected through EU harmonized monitoring for AMR in zoonotic and commensal bacteria (2013/652/EU) and UK national monitoring in 2020. Additional monitoring with an ESBL-specific agar (CHROMagar ESBL), alongside the EU-stipulated agar of MacConkey+cefotaxime, was also used for the collection of isolates in 2016 and 2018 and also for the national monitoring in 2020. DNA was extracted as previously described (Duggett et al., 2020). As mandated by EFSA and then through the UK national surveillance, a representative sampling of chicken meat samples for ESC harboring isolates occurred in alternate years (2016, 2018, and 2020) and turkey meat in 2020 using the same agar types.

### WGS and sequence analysis

All isolates were sequenced using MiSeq or NextSeq Illumina instruments, and the reads were assembled with SPAdes or Unicycler in a short-read-only mode (Bankevich et al., 2012; Wick et al., 2017). The AMR genotype was determined through the APHA SeqFinder pipeline using filters of 100% gene coverage and < 100 SNPs with respect to AMR genes in the reference database (Anjum et al., 2016). APHA SeqFinder genotypes were cross-referenced using the assembled data genotypes outputted from ABRicate using the same AMR database and a custom Python script to improve the accuracy (Seemann, 2018). The ST of the isolates was determined using multi-locus sequence typing (MLST) (Seemann, 2015). For the global comparison of isolates with the same ST as those most common in our study, matching assemblies were downloaded from Enterobase (Zhou et al., 2020). The selection of Enterobase assemblies for further phylogenetic analysis with our isolates was based on an initial neighbor-joining tree created with MashTree (Katz et al., 2019). Publicly available isolates that clustered with isolates from our study were then taken forward for whole genome alignment with Snippy using the closest genome in the GTDB r207 database as a reference, as determined by the sourmash search function (Pierce et al., 2019; Seemann, 2019). Phylogenetic trees were built using RAxML-NG with the GTR model and single nucleotide polymorphism (SNP) distances computed by snp-dists using the whole genome alignments (Seemann et al., 2018; Kozlov et al., 2019). Data were analyzed using the R collection of packages within “Tidyverse”; figures were produced using the “ggplot2” package; and co-occurrence analysis was performed using the “co-occur” package and trees visualized with ggtree after being midpoint rooted with phangorn (Yu et al., 2017; Wickham et al., 2019). All short-read data produced in this study have been deposited in the European Nucleotide Archive under PRJEB67810.

## Results

### Detection of multiple ESC genotypes

Most of the 761 ESCs harboring *E. coli* collected through the surveillance of poultry caecal or meat originated from chickens (*n* = 662). Each year, surveillance of chicken caecal samples collected from UK farms yielded fewer ESC isolates (2016; *n* = 178, 2018; *n* = 41 and 2020; *n* = 34) than UK chicken meat (2016; *n* = 241, 2018; *n* = 73 and 2020; *n* = 95) (Supplementary Table S1). Isolates recovered from turkey caecal samples also decreased each year (2016; *n* = 26, 2018; *n* = 17 and 2020 *n* = 7). Isolates from turkey meat were only collected in 2020, when it was included for monitoring, and yielded 49 ESC harboring *E. coli*, which was fewer than what was recovered from chicken meat through surveillance.

**Figure 1:**
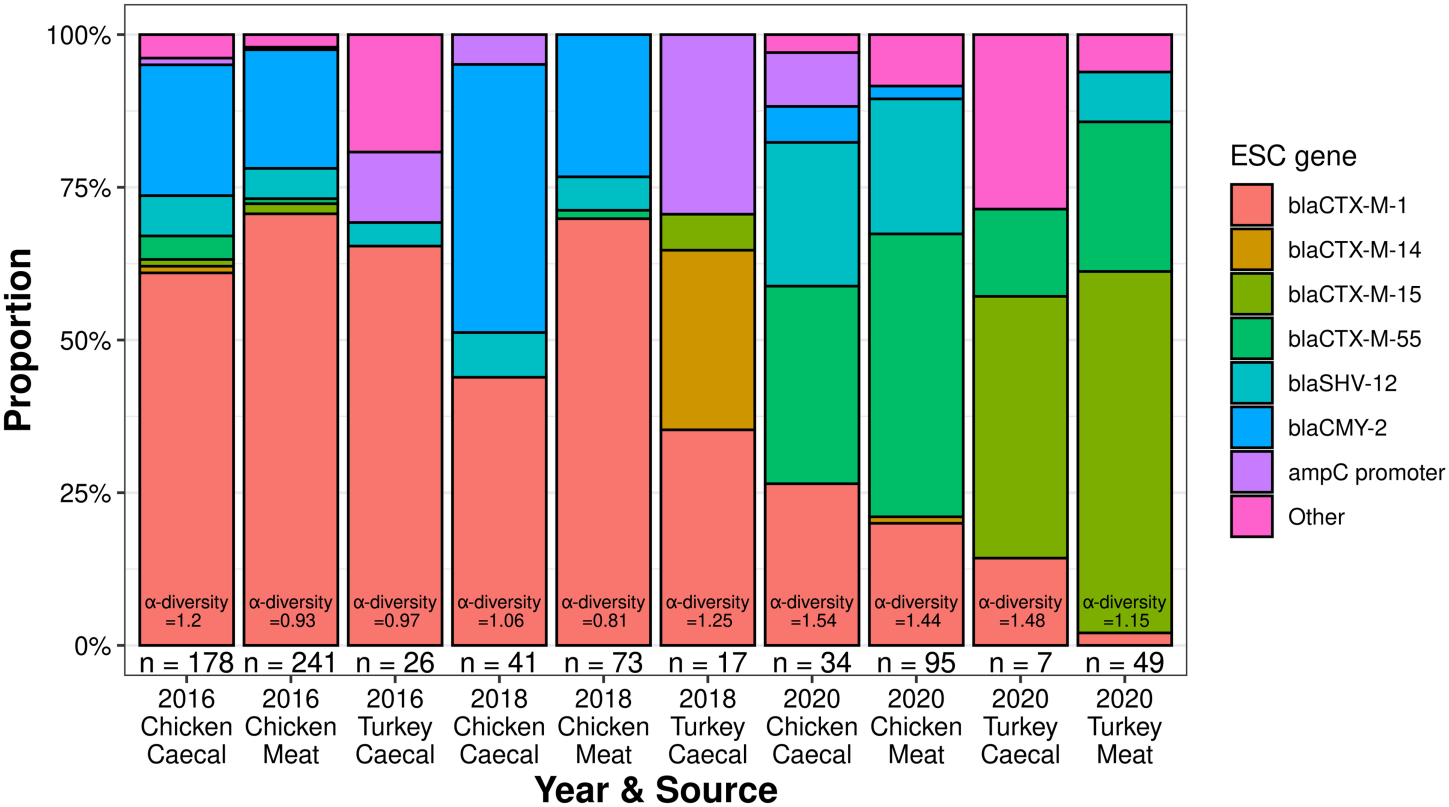
The extended-spectrum cephalosporinase proportions and diversity of poultry isolates collected from 2016 to 2020.

Multiple ESC genotypes were identified, with the most unique ESC genes identified in 2016 chicken caeca (*n* = 10). However, the 2020 chicken caeca samples had the greatest level of ESC α-diversity (Shannon-Wiener index value = 1.54, Figure 1), while the lowest level of ESC α-diversity was recovered in the 2018 chicken meat samples (Shannon-Wiener index value = 0.81). The fewest unique ESC genes were identified in the 2016 turkey surveillance and 2018 surveillance (*n* = 4). Isolates harboring *bla*_CTX-M-1_ were recovered most frequently from both sources (caecal *n* = 162 and meat *n* = 242) and formed the greatest proportion of ESC genes in 2016 and 2018 meat samples derived from chickens, which was in equal proportion to *bla*_CMY-2_ present in 2018 caecal isolates (Figure 1). However, from 2020 surveillance, *bla*_CTX-M-55_ was identified in the majority of chicken-derived isolates (*n* = 68) and *bla*_CTX-M-15_ from turkey-derived isolates.

### Genes, genotypes, and sequence types

There were 80 distinct AMR genes identified across the 761 isolates, conferring resistance to 12 classes of antimicrobials. There were 16 ESC types identified in addition to two novel SHV-12-like genes (Genbank accessions OP762696 and OP762697). The most common AMR genes were *sul2* (*n* = 521), *bla*_CTX-M-1_ (*n* = 404), and *tetA*-v4 (*n* = 385) and 17 genes were singletons. The 2016 chicken caecal isolates yielded the greatest number of unique genes (*n* = 52); however, the 2020 chicken meat survey had the most α-diversity in the AMR gene (Shannon-Wiener index value = 3.2; Supplementary Table S1). Singleton AMR genotypes formed the majority of the 216 genotypes identified, accounting for 60–83% of the genotypes found in each surveillance. The most dominant genotypes accounted for 19–40% of the total isolates recovered for each year and from the type of surveillance. These dominant genotypes were the same for chicken caeca and meat in 2016 (*bla*_CTX-M-1_, *sul2*, and *tetA*-v4) and 2020 (*aadA2*, *ant3-Ia*, *strA*, *strB*, *bla*_CTX-M-55_, *cmlA1*, *floR*, *gyrA*, *sul2*, *sul3*-v1, *tetA*-v6, and *dfrA14*) but differed in 2018 (Supplementary Table S1). For the turkey surveillance, there were no shared AMR genotypes between caeca and meat in 2020, the only year both sample types were collected.

The isolates were assigned to 105 STs, including five novel STs and 38 singletons. The most unique STs were recovered from the 2016 chicken meat surveillance (*n* = 46), and this shared the greatest level of ST α-diversity (Shannon-Wiener = 3.1; Supplementary Table S1) with the 2016 chicken caecal surveillance. Chicken caecal and meat samples shared the most common ST in 2018, which was ST57, and again in 2020, which was ST752. However, this was not the same from different sample types in 2016 chickens and in 2020 turkeys. There was a significant correlation (*p* = <0.05) between some STs and the ESC genes they harbored. For example, *bla*_CTX-M-1_ was associated with STs 38, 57, 117, 350, 602, 1,551, and 8,070; *bla*_CTX-M-15_ with ST1163; *bla*_CTX-M-55_ with ST752 and ST1640; *bla*_CMY-2_ with STs 373, 1,594, 2040, and 4,994; and *bla*_SHV-12_ with ST10 and ST117. Isolates with *bla*_CTX-M-14_ or ampC promoter mutations did not have a significant association with any ST.

### Common ST and AMR genotype combinations

**Figure 2:**
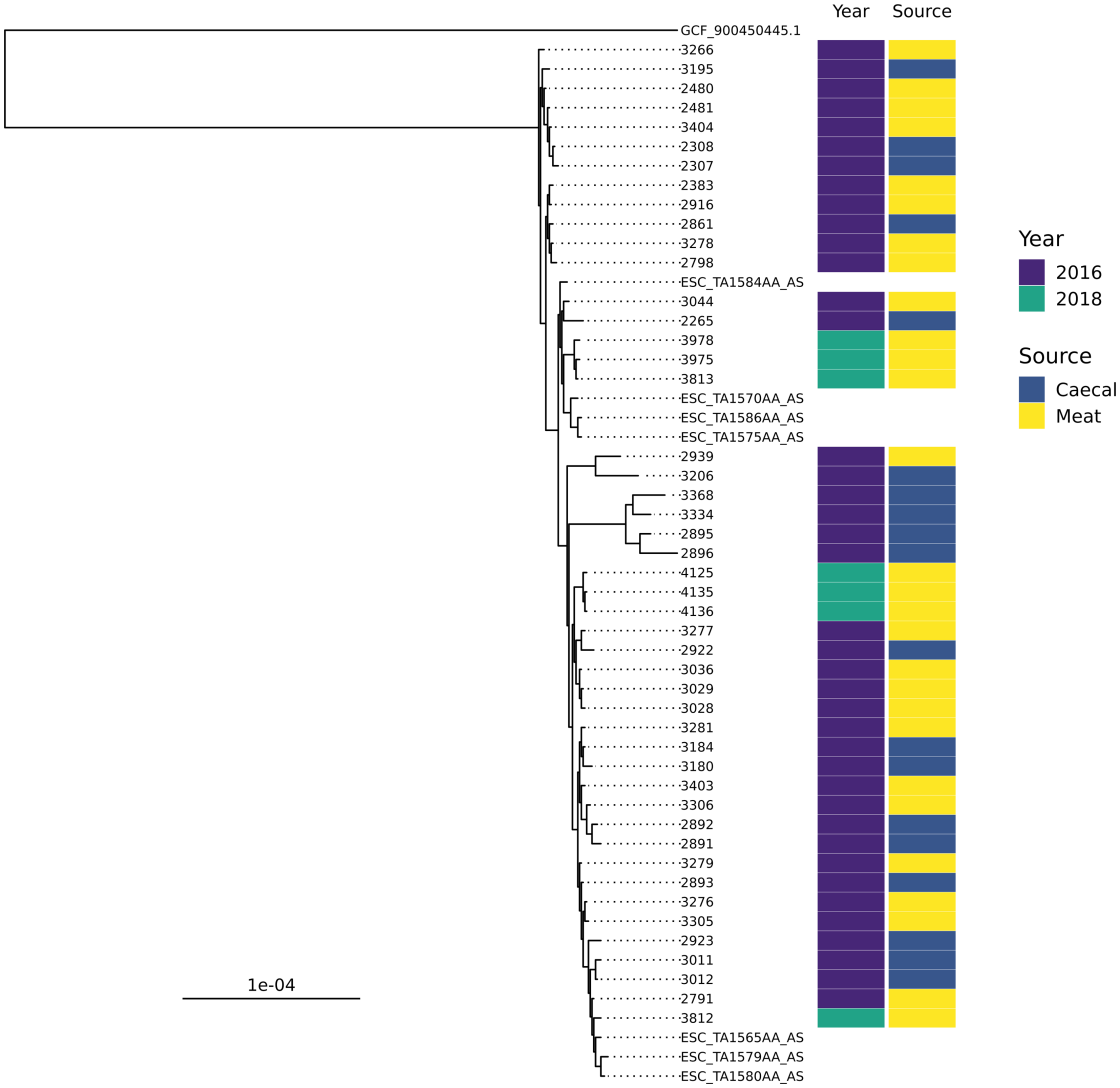
An SNP-based phylogenetic tree of ST350 isolates found in the study and those closest that were publicly available.

There were ten groups of isolates with the same AMR genotype and ST combinations identified; nine of these were found across multiple years or sample types (Table 1). The most common combination was ST350 harboring *bla*_CTX-M-1_, *sul2,* and *tetA*-v4 (*n* = 48), which was predominantly found in chickens in 2016 but was also recovered in 2018 and 2020. The AMR genes harbored by these isolates were most often found on contigs that contained an IncI1 rep-gene where sufficient genome assembly was possible. A Mashtree comparison with 84 publicly available ST350s demonstrated most isolates from our study clustered together (Supplementary Figure S1). There were seven isolates from another UK study (PRJEB21015; unpublished) that were placed nearby and so were taken forward for whole genome alignment. An ST350 isolate from our surveillance in 2020 was distinct from the other isolates and was removed from the alignment to increase tree resolution. An SNP-based phylogeny using GCF_900450445.1 as the reference showed our isolates split into three main clades (Figure 2). The phylogenetic tree showed isolates from the same year were grouped together rather than by source. An SNP-distance comparison showed that 9–125 was the lowest number of SNPs among the isolates (median = 17), indicating a high level of similarity between isolates. Two sets of two isolates were clonal as they were ≤ 10 SNPs apart (Storey et al., 2022) with all four originating from 2016 chicken meat. This AMR genotype was also commonly identified in ST38 (*n* = 39), ST57 (*n* = 29), and ST117 (*n* = 25).

**Table 1:** Most common MLST+AMR genotype combinations from 2016–2020 poultry isolates collected in this study.

Sequence type	Genotype	Years identified	Animal	Source	Number of isolates
350	*bla*_CTX-M-1_, *sul2*, and *tetA-v4*	2016	Chicken	Caecal	48
				Meat	
		2018			
		2020			
752	*aadA2, ant3-Ia, strA, strB*, *bla*_CTX-M-55_, *cmlA1, floR, gyrA, sul2, sul3, tetA-v6,* and *dfrA14*	2020	Chicken	Caecal	40
				Meat	
38	*bla*_CTX-M-1_, *sul2,* and *tetA-v4*	2016	Chicken	Caecal	39
				Meat	
602	*aadA5, strA, strB, bla*_CTX-M-1_*, sul2, tetA(B), dfrA17,* and *fosA7.5*	2016	Chicken	Caecal	35
				Meat	
			Turkey	Caecal	
2040	*bla*_CMY-2_	2016	Chicken	Caecal	31
				Meat	
		2018		Caecal	
				Meat	
		2020		Caecal	
57	*bla*_CTX-M-1_, *sul2,* and *tetA-v4*	2018	Chicken	Caecal	29
				Meat	
		2020		Caecal	
				Meat	
117	*bla*_CTX-M-1_, *sul2,* and *tetA-v4*	2016	Chicken	Caecal	25
				Meat	
		2018			
		2020			
1,163	*bla*_CTX-M-15_*, gyrA, qnrS1,* and *tetA-v4*	2020	Turkey	Meat	18
602	*aac3-IVa, aadA5, ant3-Ia, strA, strB, aph4-Ia, bla*_CTX-M-1_*, mphB, sul1, sul2, tetA(B),* and *dfrA1, dfrA17,* and *fosA7.5*	2016	Chicken	Caecal	12
				Meat	
4,994	*bla*_CMY-2_ and *gyrA*	2016	Chicken	Caecal	10
				Meat	
		2018			

**Figure 3:**
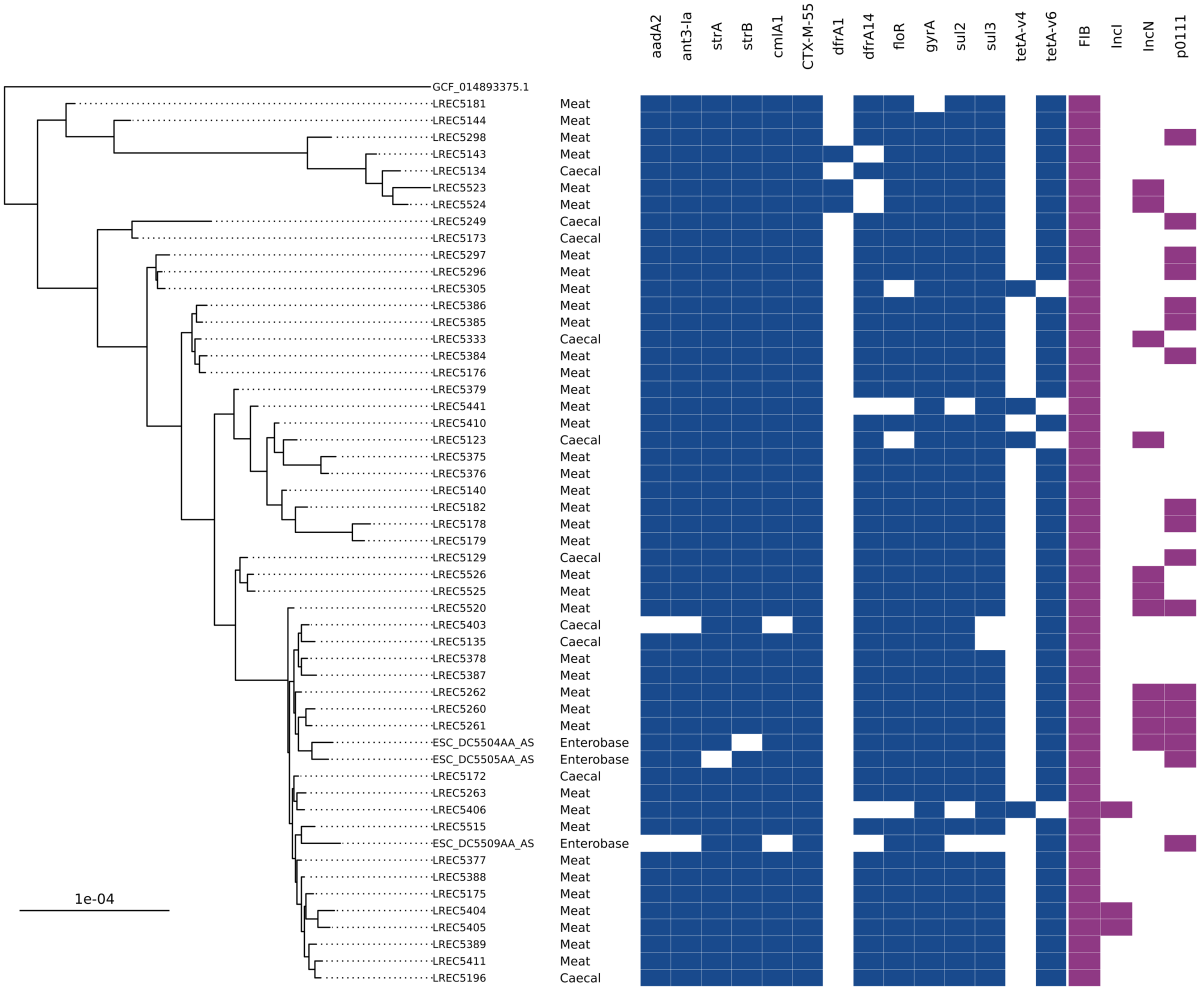
An SNP-based phylogenetic tree of ST752 isolates found in the study and those closest that were publicly available.

A group of ST752 *bla*_CTX-M-55_ isolates harboring resistance to nine antimicrobials (12 unique genes) was the most common AMR genotype overall in 2020 chicken isolates. They accounted for 24% of caecal isolates (*n* = 7) and 35% of meat isolates (*n* = 33) and were only isolated in the second half of 2020 (Table 1). There were a further 10 ST752 *bla*_CTX-M-55_ harboring isolates recovered from caeca or meat in 2020 that had a subset of these genes or, in three cases, a single allelic difference in *dfrA*. Hybrid-assembly of one isolate revealed that it harbored three genes (*tetA*, *strA*, and *strB*) chromosomally that were also found with the other AMR genes on an IncFIB-IncN hybrid plasmid that was 140 kb with low overall sequence similarity to any other plasmid in the National Center for Biotechnology Information (NCBI; 79% query coverage; MF589339.1). Phylogenetic comparison between the 315 ST752s on Enterobase using Mashtree showed three isolates submitted to Enterobase by the Scottish *Salmonella*, *Shigella,* and *C. difficile* Reference Laboratory (SSSCDRL) in 2022 clustered with our isolates and shared similar AMR genotypes; these isolates were taken forward for whole genome alignment (Figure 3). SNP-distance analysis showed the lowest number of SNPs as 31, with a median of 48 and a range of 31–125. The closest isolates from our dataset to those from SSSCDRL were 82 SNPs apart.

ST1163 harboring CTX-M-15, *gyrA*, *qnrS1*, and *tetA*-v4 was only identified in a single surveillance set (2020 turkey meat) and was recovered 18 times, comprising 37% of all isolates collected during this survey. There were two further ST1163 isolates harboring additional AMR genes. Hybrid-assembly of one of the ST1163 isolates showed that the acquired resistance genes were on a ~ 22.5 kb segment integrated into the chromosome with 100% identity to fragment on an IncY plasmid in NCBI (p17-AB00432: MT158480.1). The ST1163 isolates were only collected in the last quarter of 2020 and the number of SNPs between isolates from our study ranged from 11–67 with a median of 37. Comparison with ST1163 isolates from Enterobase (*n* = 84) yielded no isolates on the same branches, indicating a limited similarity between these groups. However, the closest isolate was 34 SNPs apart and collected in the UK from a human in 2021 (SRR169410/ESC_AC5982AA; Supplementary Figure S2).

**Figure 4:**
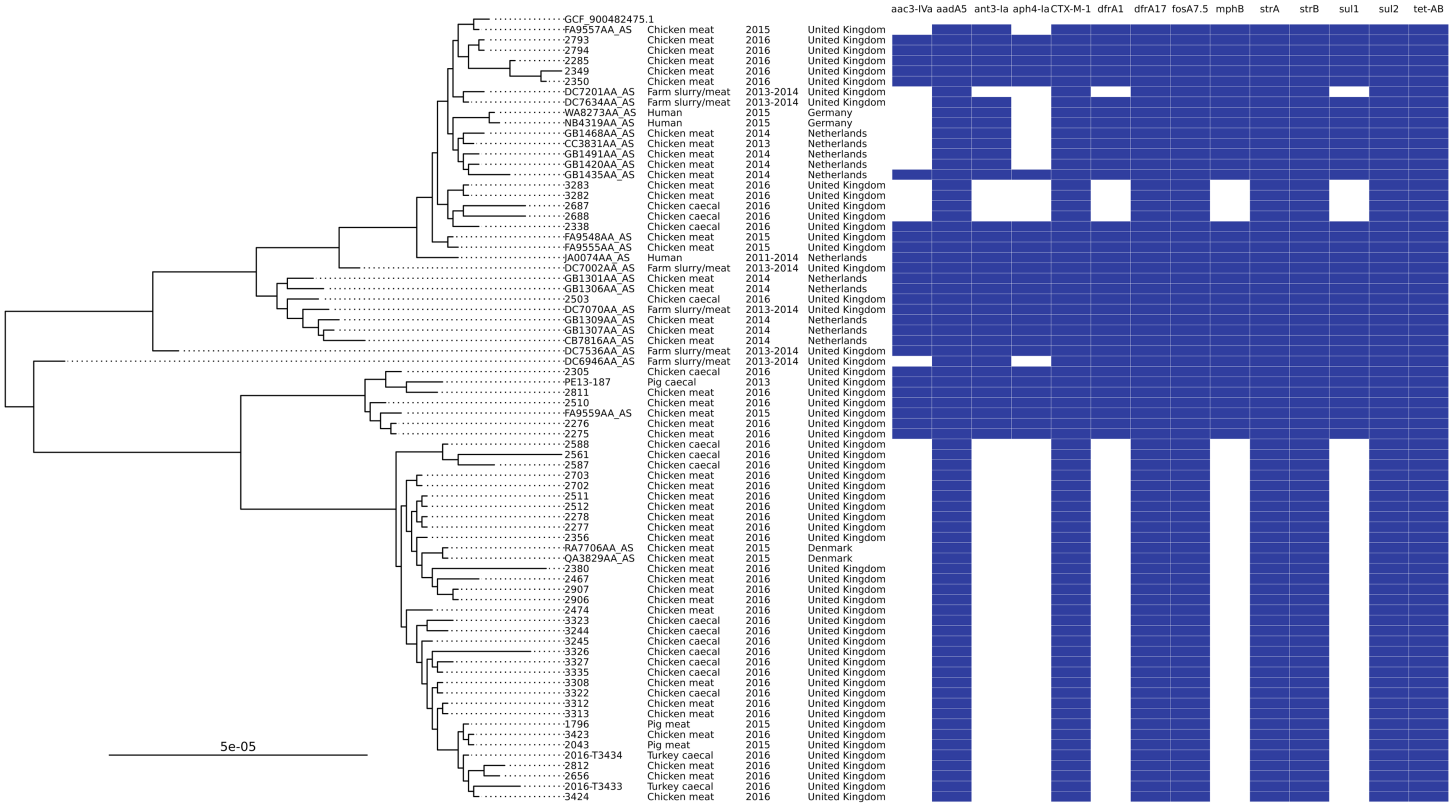
An SNP-based phylogenetic tree of ST602 isolates found in the study and those closest that were publicly available.

Searching the most common AMR genotype and ST combinations in poultry against isolates recovered from UK pigs in 2013–2019 (Duggett et al., 2020) yielded three isolates that matched. These isolates were ST602, with two isolated from 2015 pork samples of UK origin and one from a 2013 pig caecal sample. This group of ST602s harbored IncI1 plasmids that contained *aadA5*, *bla*_CTX-M-1_, *sul2*, and *dfrA17* in addition to IncFIB or IncFIC plasmids with *strAB*. A Mashtree comparison of all Enterobase ST602s (*n* = 485) showed that there were 70 isolates that clustered on the same branches or directly next to the isolates reported in this study, but further SNP analysis showed that 45 of these were on a distal clade; as a result, they were removed from the final phylogenetic tree (Figure 4). The tree showed the isolates split into two main clades, and some neighboring isolates shared the same AMR genotype. Isolates from Denmark, Germany, and the Netherlands were clustered with isolates from our study, in addition to those from other UK studies. Most isolates originated from chicken meat, although three were from humans (two from Germany and one from the Netherlands). Owing to limited metadata for isolates from NCBI, the exact isolation date was uncertain, but they were isolated between 2011 and 2016. The isolates from 2015 UK pork clustered with those from 2016 chicken meat and SNP-distance analysis showed that these isolates were 2–4 SNPs apart, indicating that the same clone was circulating. The 2013 pig caecal isolate was placed next to a chicken meat isolate but was found to have 56 SNPs different, thereby being much less related. The lowest number of SNPs between isolates was 2–91, with a median of 24.

A single ST131 O16:H5 fimH41 isolate was recovered that harbored *bla*_CTX-M-15_, *bla*_TEM-1b_, *aadA5*, *mphA*, *sul1*, and *dfrA17*. Although this isolate from the 2020 turkey meat survey harbored an IncFIB plasmid, the fragmented nature of short-read assemblies meant that the AMR genes could not be co-linked. On comparison of its genome to the 14,333 ST131 isolates on Enterobase, it clustered equally close to two isolates, one from a human in Lebanon collected in 2018/2019 and one from Germany without metadata. Both isolates were 69 SNPs different from our strain when using a whole genome alignment to the closest reference isolate, NZ_CP015159.1.

### Linkage of ESCs with other AMR genes and plasmids

The co-occurrence of ESCs and other AMR genes was identified multiple times in our data. For the ESCs that occurred more than 30 times, *bla*_CTX-M-55_ had the greatest number of significantly associated AMR genes (*p* = <0.05; *n* = 11) with *bla*_CTX-M-15_ and *bla*_CMY-2_ having the fewest (*n* = 3; Supplementary Table S2). There were 11 plasmid-types co-linked with AMR genes. The plasmid type with the most AMR genes associated was IncI1, which had 19 unique genes associated with it; *bla*_CTX-M-1_ (*n* = 165) being the most prevalent (Supplementary Table S3). IncI1 also had other ESCs present, such as *bla*_CMY-2_, *bla*_SHV-12,_ and *bla*_TEM-52c_. Other plasmid-types that had ESCs on the same contig included IncBOKZ with *bla*_CMY-2_ (*n* = 30), IncFIB with *bla*_CMY-2_ (*n* = 3), *bla*_CTX-M-1_ (*n* = 7) and *bla*_CTX-M-15_ (*n* = 3), IncX1 with *bla*_CTX-M-1_ (*n* = 1) and *bla*_TEM52-b_ (*n* = 2), and p0111 with *bla*_TEM52-c_ (*n* = 5). However, there was no significant association between any of the plasmid replicon and AMR genes, probably due to the short-read assemblies being too fragmented.

### Trends for co-resistances in chicken caeca and meat samples

Genotypic co-resistance to non-beta lactam antimicrobials in the EFSA panel (Duggett et al., 2021) was common, and both sample types had the same median of resistances for each year (Figure 3). Multidrug resistance (MDR), as determined by resistance to three or more classes of antimicrobials (AbuOun et al., 2021), was a common attribute (61–97.9% of isolates) and, while the level fluctuated between 2016 and 2020 in both sample types, isolates derived from meat had higher proportions of MDR in each year (Supplementary Table S1). The greatest number of resistances in a single isolate was nine, which was recovered from the 2018 meat surveillance. Across all years, co-resistance was most common to sulphamethoxazole (56–89%), tetracycline (54–85%), and trimethoprim (15–63%; Supplementary Figure S3). Isolates recovered in 2020 also frequently harbored resistance to chloramphenicol (caecal 50% and meat 65%) and ciprofloxacin/nalidixic acid (caecal 50% and meat 61%), which was higher than that observed in other years. Genes conferring resistance to the HP-CIAs azithromycin (*mph*A) and colistin (*mcr*-9) were identified in single isolates from chicken meat in 2020. Resistance to a non-EFSA panel of antimicrobials was detected in years and sources, including critically important erythromycin, kanamycin, fosfomycin, and rifampicin; highly important lincomycin, and important spectinomycin.

### Characterization of isolates derived from turkey AMR surveillance

**Figure 5:**
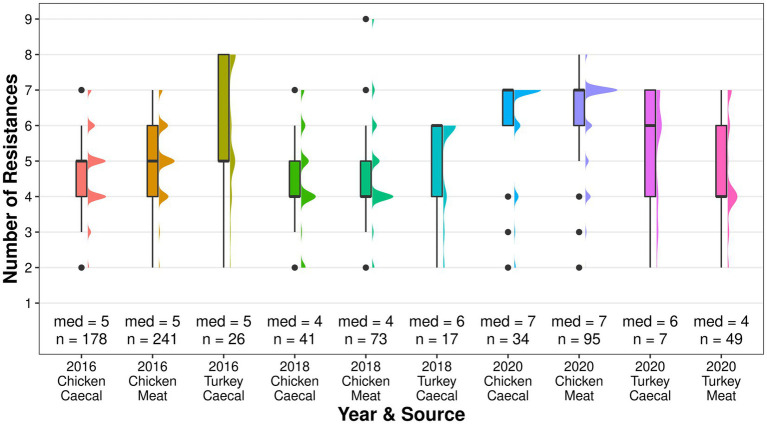
The median number of genotypic resistances identified in poultry isolates collected through surveillance of AMR in caecal and meat from 2016 to 2020.

Although *bla*_CTX-M-1_ was dominant in 2016, it became less common with each year of surveillance with *bla*_CTX-M-15_ becoming the dominant ESC in 2020 (Figure 1). The median resistance of the turkey isolates fluctuated and was comparable to chickens in 2016, higher in 2018, and lower in 2020 (Figure 5). Co-resistance was most common to sulphamethoxazole in 2016 and 2018 but resistance to ciprofloxacin/nalidixic acid was most common in 2020. MDR was common and fluctuated between 76.5% in 2018 and 85.7% in 2020 in the caecal isolates; 79.6% of the meat isolates were MDR.

## Discussion

Genomic surveillance of ESC isolates collected from national monitoring yields a plethora of data beyond that available through classical microbiology or other molecular methods such as PCR. Specifically, the monitoring of caecal and meat isolates in poultry enables the determination of the overlap between the two sample types and the tracking of isolates through the food production system at a deeper level. To quantify the difference and view the trends in the ESC genes recovered in each group of isolates, the α-diversity was calculated. While a comprehensive understanding of the mechanisms driving differences in diversity may still need to be established, it provides a numerical value to the year-on-year changes we see beyond the summary graphs previously published (Duggett et al., 2021). Unlike the isolates recovered from the corresponding study of UK pig caecal samples from 2013 to 2019, (Duggett et al., 2021), the poultry isolates showed a much greater diversity of STs for each AMR genotype. There were 10 ST and AMR genotype groups detected, and together, these accounted for 38% of all isolates collected through surveillance in this period. Comparison with publicly available isolates demonstrated limited similarity, with only a handful of clustering with ones from this study intimating that these are UK-specific variants. Comparing SNP distances of isolates with the same ST and AMR genotype showed that the majority were not clonal (<14 SNPs apart) (Storey et al., 2022) but closely related. This suggests that the isolates likely originated from the same progenitor. While the recovery of the same ST/AMR genotype combination did occur between subsequent surveillance years, it was often heavily weighted to single years, which implies a boom-and-bust scenario for successful ST/AMR combinations which dominate before being replaced by new combinations. However, as poultry surveillance is performed bi-annually, replacement with new combinations may be longer than annual but not evident in our data, and there may be other factors than just the introduction of new bird flocks onto farms, bringing with them their own combinations.

There was a reduction in the number of ESC isolates recovered from poultry caeca from the UK from 2016 to 2020 (*n* = 178 to *n* = 34). However, despite an initial drop in the number of chicken meat ESC isolates in 2016, there was an increase from 2018 to 2020 (*n* = 73 to *n* = 95). Also, the overall AMR numbers per isolate from poultry caeca and chicken meat fell from 2016 to 2018, but there was a marked increase in the median number of AMR genes in 2020 in isolates from the same source. This was primarily driven by the high numbers of ST752s recovered that harbored resistance to nine antimicrobials. This group of isolates also marked a change in the dominant ESC from *bla*_CTX-M-1_ to *bla*_CTX-M-55_ in chickens. In turkeys, the number of isolates dropped each surveillance year for caeca. The inclusion of turkey meat for 2020 showed most isolates were ST1163 harboring *bla*_CTX-M-15_, but they showed no similarity to publicly available ST1163 isolates.

The persistence of MDR ESC isolates in livestock and food may be driven by the use of other antimicrobials that co-select, as the British Poultry Council (BPC) advised farmers in 2012 that cephalosporins are no longer to be used for poultry meat production. In 2016–2020 members of the BPC, antibiotic stewardship most commonly used amoxicillin (44–71%) and tetracyclines (12–38%) (VMD, 2016, 2019, 2021). Resistance to amoxicillin was present in all ESC isolates due to their nature, but resistance to tetracycline was also frequently occurring in 14–85% of isolates in each survey, including in eight of the ten most common AMR genotype and ST combinations.

Identification of common genotypes, sometimes with additional genes but in different STs, further alludes to circulating ESC MDR plasmids, which indicates that these plasmids can be readily disseminated to a range of STs. In one dataset, ST602 isolates from different One Health compartments, including livestock, meat, and humans (in mainland Europe), were linked multiple times. While these isolates may not harbor pathogenic genes, the potential dissemination of these MDR plasmids to pathogens will hamper downstream treatment from infections. Further study to compare the plasmids found in these compartments with those from humans is required to determine how widespread it may be, although selected analyses have suggested that these links exist (Duggett et al., 2021; Matlock et al., 2023).

There are some limitations to our study, for example, as there are alternative years of collection between pigs and poultry for the national AMR surveillance as this makes it challenging to track the groups of isolates with the same ST and AMR genotype beyond each collection year. Furthermore, the anonymity of the sample collection prohibits targeted visits to farms to trace the source of the closely related isolates. From our study, it is difficult to ascertain whether the lack of similarity to publicly available isolates is due to the paucity of veterinary genomic surveillance compared to humans or whether we have identified UK-specific variants. The UK already carries out WGS as a matter of routine on suspect ESCs isolated from selective plates and has done since 2015, which is published annually. Genomic surveillance of ESC isolates has become optional from 2021 and will hopefully result in an increase of WGS for EU member states. If these data become publicly available, this will offer valuable data and provide the basis for comparisons of neighboring countries, potentially identifying the most successful STs, plasmids, or emerging pathogenic threats, which will also provide vital information for policymakers and others to identify risk pathways and interventions to reduce the global AMR burden.

## Data availability statement

The datasets presented in this study can be found in online repositories. The names of the repository/repositories and accession number(s) can be found below: EBI, PRJEB67810 www.ebi.ac.uk/ena/browser/view/PRJEB67810.

## Author contributions

ND: Conceptualization, Data curation, Formal analysis, Investigation, Methodology, Validation, Visualization, Writing – original draft, Writing – review & editing. MA: Conceptualization, Data curation, Writing – review & editing. ES: Data curation, Writing – review & editing. OT: Data curation, Writing – review & editing. LR: Data curation, Writing – review & editing. RH: Writing – review & editing. JN-G: Data curation, Methodology, Software, Writing – review & editing. DG: Data curation, Writing – review & editing. JC: Data curation, Writing – review & editing. CT: Conceptualization, Funding acquisition, Supervision, Writing – review & editing. MFA: Funding acquisition, Project administration, Supervision, Writing – review & editing.
