# Supplementary figures and images for: Genomic surveillance of extended-spectrum cephalosporin-resistant Escherichia coli isolated from poultry in the UK from 2016 to 2020
Source: Front Microbiol. 2024 Jan 30;14:1335173. doi: 10.3389/fmicb.2023.1335173 (PMC10861728; doi:10.3389/fmicb.2023.1335173)

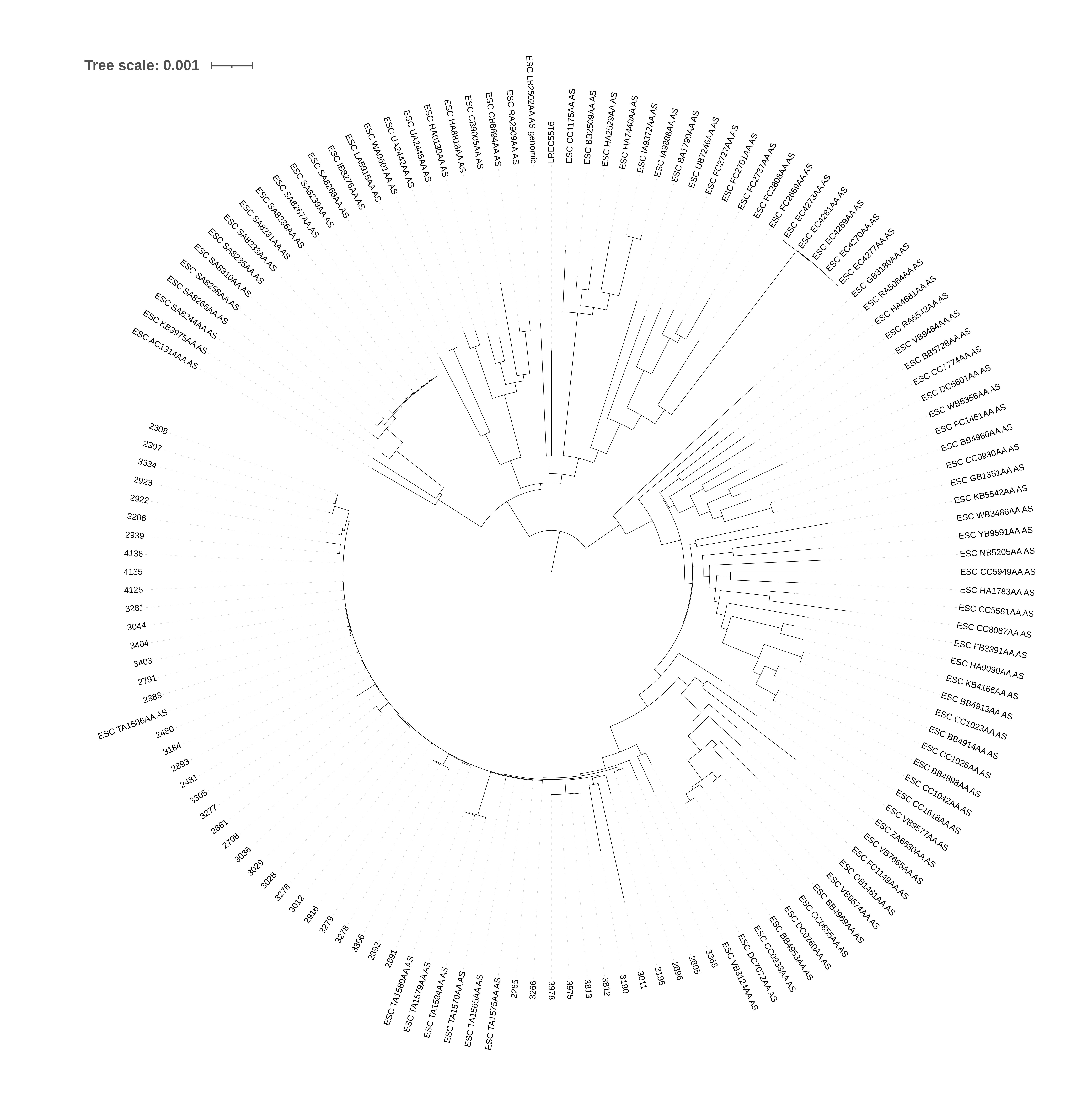

Supplement: Supplementary file 1 [file Image_1.JPEG]

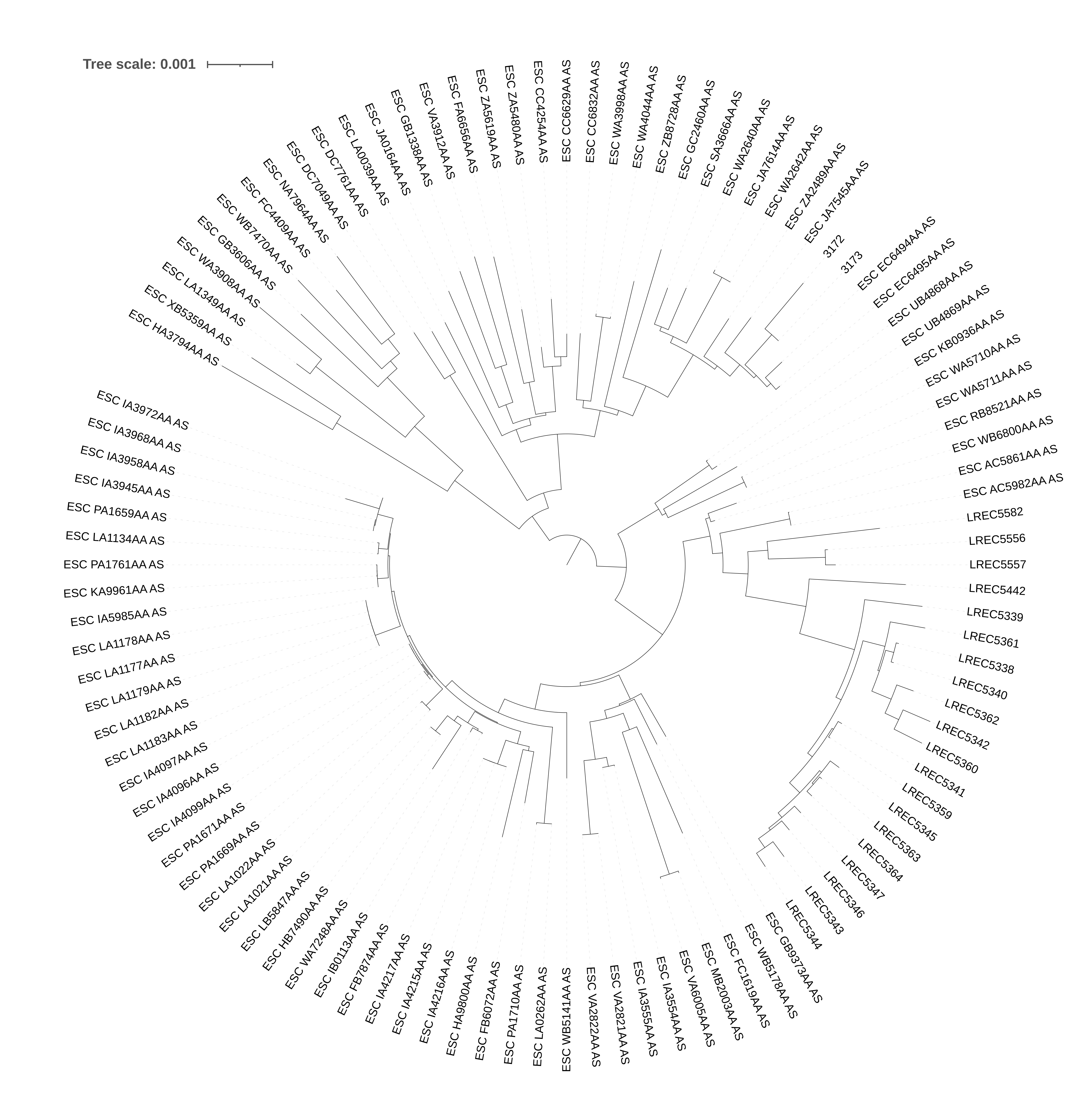

Supplement: Supplementary file 2 [file Image_2.JPEG]
